# Targeting interleukin-1 signaling for renoprotection

**DOI:** 10.3389/fimmu.2025.1591197

**Published:** 2025-05-23

**Authors:** Daria Bogdanova, Mikhail Y. Samsonov, Svetlana Lebedeva, Darya Bukhanova, Maria Materenchuk, Kerim Mutig

**Affiliations:** ^1^ Division of Immunobiology and Biomedicine, Sirius University of Science and Technology, Sochi, Russia; ^2^ Institute of Cytology RAS, St. Petersburg, Russia; ^3^ Medical Department, R-Pharm JSC, Moscow, Russia; ^4^ Department of Pharmacology, Institute of Pharmacy, I.M. Sechenov First Moscow State Medical University (Sechenov University), Moscow, Russia

**Keywords:** cytokines, chronic kidney disease, acute kidney injury, interleukins, nephroprotection, ADPKD (autosomal dominant polycystic kidney disease)

## Abstract

Sterile inflammation with ensuing immune-mediated kidney damage has been implicated in pathophysiology of acute and chronic kidney diseases. Disinhibition of interleukin 1 (IL-1) signaling triggers local inflammation of renal tissue and may initiate or aggravate systemic inflammatory response. The IL-1α isoform is released by many cell types during cell necrosis to attract immune cells, whereas the IL-1β isoform is secreted by immune cells to amplify local inflammatory responses. The unfolding of IL-1 signaling is restricted by an endogenous IL-1 receptor antagonist and a decoy IL-1 receptor variant. Pharmacological IL-1 inhibitors mimicking the natural IL-1 suppressors are instrumental in management of a broad spectrum of (auto)inflammatory disorders. Progression of several kidney diseases toward renal fibrosis has been associated with a disbalance between the pro-inflammatory and anti-inflammatory IL-1 signaling components. While IL-1 inhibitors have proven success in prevention and treatment of renal complications accompanying the autoimmune disorders, broader opportunities in kidney diseases have been expected. The present review work analyzes potential niches for IL-1 signaling in the field of nephrology.

## Interleukin 1 family

**Figure 1:**
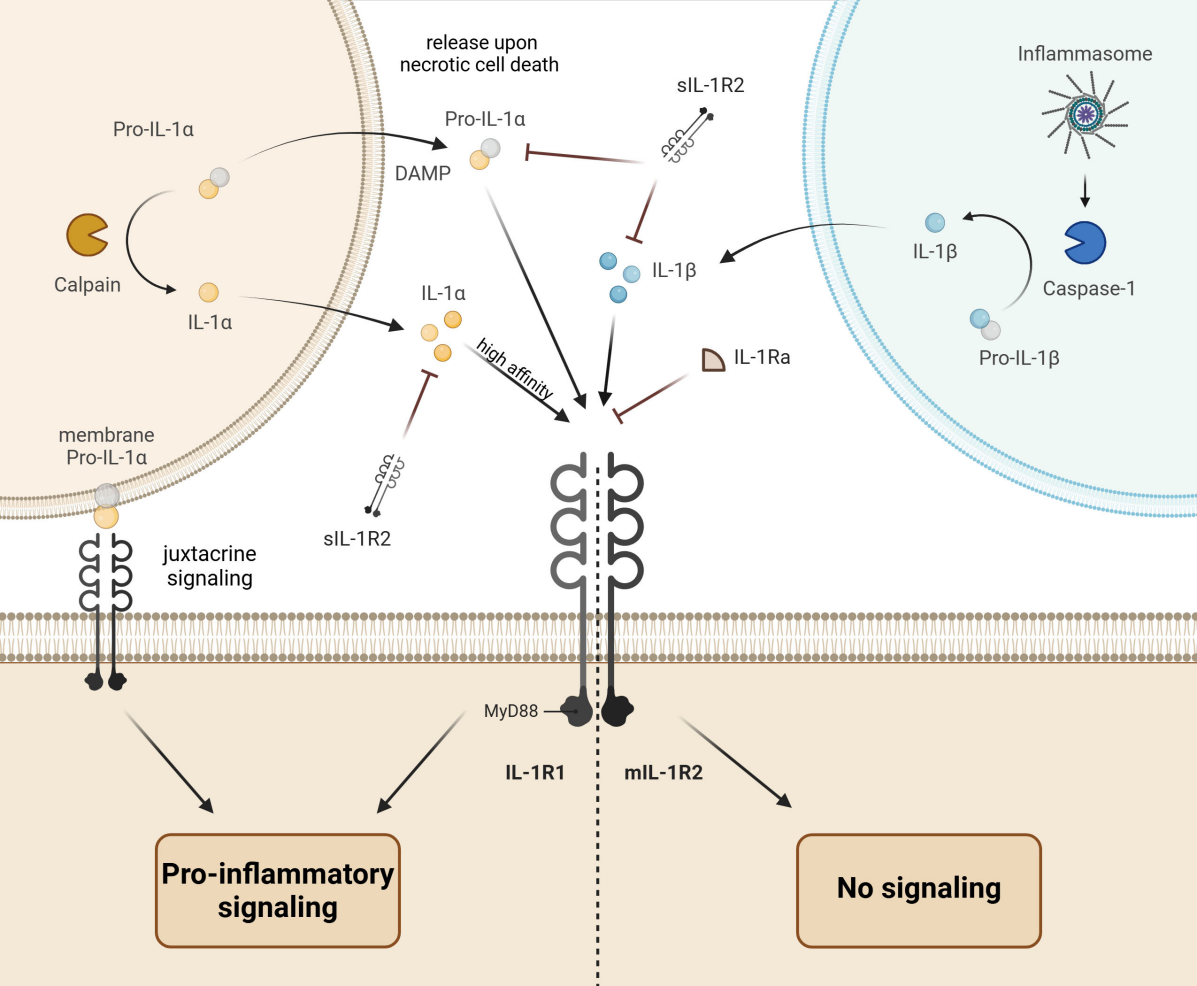
Schematic drawing of interleukin 1 (IL-1) signaling. The IL-1α isoform precursor (pro-IL-1α) is produced in epithelial, endothelial, or glial cells and released upon necrotic cell death to induce the pro-inflammatory response in neighboring immune cells via binding to the IL-1 receptor type 1 (IL-1R1) and downstream signal transduction through the myeloid differentiation primary response 88 adaptor protein (MyD88). The pro-IL-1α can be further presented at the cell surface to the neighboring IL-1R1-expressing cell (juxtacrine signaling). Finally, activation of calpains (Ca^2+^-dependent proteases) during necrotic cell death may lead to proteolytic cleavage and release of mature IL-1a with higher affinity to IL-1R1. In contrast to the active pro-IL-1α, the precursor of IL-1β isoform (pro-IL-1β) produced by immune cells is inactive. The proteolytic cleavage of pro-IL-1β in inflammasomes by caspase-1 converts it to the active IL-1β, which induces and amplifies the inflammatory response upon release and binding to IL-1R1. The pro-inflammatory action of IL-1α and IL-1β is balanced by endogenous inhibitors encompassing the IL-1 receptor antagonist (IL-1Ra) and the decoy type 2 IL-1 receptor existing in the membrane bound (mIL-1R2) and soluble forms (sIL-1R2).

Members of the interleukin 1 (IL-1) family coordinate the innate and adaptive immunity thus enabling the proper functioning of the immune system (1). The IL-1 family comprises 11 cytokines endowed with agonistic (IL-1a, IL-1b, IL-18, IL-33, IL-36a, IL-36b, and IL-36g), antagonistic (IL-1Ra, IL-36Ra, and IL-38), or anti-inflammatory activities (IL-37) mediated by 10 specific receptors (2, 3). The receptors possess the Toll-IL-1 resistance (TIR) domain enabling their dimerization upon ligand binding, followed by signal transduction via recruitment of the myeloid differentiation primary response 88 adaptor protein (MyD88) connecting the downstream kinases to the dimerized receptors. The ensuing activation of major kinase and transcription signaling pathways including the c-Jun N-terminal kinase (JNK), mitogen-associated protein kinases (MAPKs), extracellular signal-regulated kinases (ERKs), nuclear factor-κB (NF-κB), activator protein-1 (AP-1), and interferon-regulatory factors (IRF) elicits differential cell-type specific effects. While all IL-1 members signal via MyD88, effects of distinct cytokines are largely determined by cell-type specific receptor repertoires. A fine-tuning of IL-1 family cytokine signaling by differentially expressed endogenous suppressors is another major player in response specificity (2). A complex network of IL-1 cytokines with agonistic, antagonistic, or anti-inflammatory activities, their signal-transducing or decoy receptors, as well as intracellular negative regulators fulfils differential tasks in coordinating the innate and adaptive immunity. Thus, functional interactions of IL-1 isoforms (IL-1α or IL-1β) with their cognate type 1 receptor (IL-1R1) are modulated by endogenous competitive inhibitors encompassing the IL-1 receptor antagonist (IL-1Ra) and the decoy type 2 receptor existing in the membrane-bound and soluble variants (mIL-1R2 and sIL-1R2), as specified below and in **Figure 1**.

## IL-1α vs. IL-1β

IL-1 was identified by early studies on leukocyte-derived pyrogens as a factor comprising two molecules, which was confirmed by later cDNA cloning of the IL-1α and IL-1β isoforms (4). Although both IL-1 isoforms share the same receptor for signal transduction, differences in their expression patters, activity regulation, and cellular sites of action enable largely non-redundant functional spectra for IL-1α vs. IL-1β. Both IL-1α and IL-1β are synthesized as intracellular precursors but the IL-1α precursor is biologically active, whereas the IL-1β precursor requires cleavage by caspase-1 for activation (2, 5, 6).

The IL-1α precursor exhibits broad and constitutive expression in epithelial, endothelial, and glial cell types with enriched presence in tissues constituting the barrier function such as keratinocytes in the skin, epithelial lining of the gastrointestinal tract, type 2 epithelial cells in the lung, urinary bladder epithelium, or astrocytes in the brain (5). Due to constitutive intracellular abundance of IL-1a in the active precursor form its release upon necrotic cell death triggers sterile tissue inflammation via IL-1R1 activation in adjacent cells. Thus, IL-1α functions as an alarmin, i.e. a component of the Damage-Associated Molecular Patterns (DAMPs) mediating recognition of cell necrosis and tissue injury by the immune system (5). Additional induction of IL-1α expression and release by injured tissue cells and resident myeloid cells in response to inflammatory stimuli further promotes infiltration of immune cells from systemic circulation. Apart from the IL-1a release, the cytokine can act as an integral membrane protein expressed on the cell surface of macrophages and capable of IL-1R1 activation on neighboring cells to induce focal inflammatory response (7). In contrast to the classical paracrine signaling, the membrane-bound IL-1α signal transduction mode is referred to as juxtacrine signaling (5, 8). The induction of pro-inflammatory IL-1α effects is tightly controlled by intrinsic mechanisms. Intracellular binding of the IL-1α precursor with the decoy IL-1R2 receptor reduces the active cytokine pool thus silencing IL-1α in case of cell necrosis (9). Similarly, nuclear translocation and binding of IL-1α with DNA during apoptosis prevents its release in the active form (10). In contrast, proteolytic processing of the IL-1α precursor (pro-IL-1α) by calpain converts the cytokine to a more potent IL-1R1 ligand, i.e. biological activity of the mature IL-1α is substantially higher compared to the pro-IL-1α (11). The intracellular buffering or proteolytic processing of pro-IL-1α adjust its pro-inflammatory impact on the environment in different modalities of regulated cell death (12).

Unlike IL-1α with its broad constitutive expression pattern and active precursor form, IL-1β is an inducible cytokine of myeloid cells produced in response to inflammatory stimuli as an inactive precursor with ensuing proteolytic processing by caspase 1 and/or caspase 11 to obtain the mature IL-1β with high affinity to its cognate receptor IL-1R1 (11, 13). The proteolytic cleavage of pro-IL-1β takes place in inflammasomes constituting cytosolic multi-molecular signaling platforms containing a nucleotide-binding oligomerization domain-like receptor (NLR), the adapter apoptosis-associated speck-like protein with a caspase recruitment domain (ASC), and the effector protease caspase-1 (14). Among different inflammasome types defined by distinct NLR proteins, the inflammasomes involving the NOD-, leucine-rich repeat (LRR)- and pyrin domain (PYD)-containing protein 3 (NLRP3, also known as NALP3) appear to play the most relevant role in the IL-1β maturation (14). The formation of NLRP3 inflammasomes occurs in two steps known as priming (Signal 1) and protein complex assembly (Signal 2). The priming process represents the transcriptional and translational response to pathogen-associated molecular patterns (PAMPs) or DAMPs mediated by the Toll-like receptor 4 (TLR4) activation or tumor necrosis factor (TNF) signaling, subsequently driving the NF-κB-dependent synthesis of NLRP3, pro-IL-1β, and pro-IL-18. The ensuing inflammasome assembly and activation involves complex cellular events induced by various PAMPs or DAMPs such as dysfunction and damage of cell organelles, oxygen species (ROS) generation, or changes in intracellular ion concentrations (14). In fact, formation and activation of NLRP3 inflammasomes is driven by microbial or sterile inflammatory stimuli and results in maturation and release of active IL-1β (15). The ensuing activation of IL-1R1 on neighboring cells triggers release of further potent inflammatory mediators including IL-6 thus amplifying the local pro-inflammatory reactions (16). The IL-1β:IL-1R1 signaling occurs via recruitment of the interleukin-1 receptor accessory protein (IL-1RAcP, also referred to as IL-R3) and activation of the downstream interleukin-1 receptor-associated kinase (IRAK) and stress-activated protein kinases (SAP) (17). The induction of IL-6 in response to IL-1β is mediated by the phosphatidylinositol 3-kinase (PI3K) and the protein kinase B (historically termed as Akt (18)) upstream of NFκB (19). Taken together, IL-1β is an inducible cytokine derived from myeloid cells such as monocytes, dendritic cells (DCs), tissue macrophages or granulocytes and exerting potent pro-inflammatory effects via IL-1R1 with ensuing induction of IL-6 and other pro-inflammatory factors.

## Interleukin-1 receptor antagonist

IL-1Ra binds to IL-1 receptors but does not elicit intracellular signaling (20). Alternative splicing of the *IL1RN* gene encoding for IL-1Ra enables its soluble (sIL-1Ra) and intracellular variants (icIL-1Ra) (20). Moreover, three intracellular isoforms (icIL-1Ra1, 2, and 3) have been identified (21). The soluble form can be secreted by monocytes, macrophages, neutrophils, hepatocytes, and microglial cells, whereas the intracellular variants are mainly expressed in keratinocytes and other epithelial cells, monocytes, macrophages, and fibroblasts (21). Functionally, sIL-1Ra exerts potent anti-inflammatory effects as a competitive blocker of IL-1β or IL-1α. The biological roles of icIL-1Ra variants remain unclear, although their release by keratinocytes has been suggested to exert anti-inflammatory effects on the microenvironment (21, 22).

## IL-1R1 vs. IL-1R2

Two IL-1 receptors, IL-1R1 and IL-1R2, originating from distinct genes have been identified (23). IL-1R1 possesses the cytoplasmic TIR domain and is capable of initiating the cellular signaling in response to IL-1β, IL-1α precursor, or mature IL-1α via formation of ternary complex with IL-R3, TIR-mediated binding of MyD88, and MyD88-mediated recruitment of IRAK4. The resulting IRAK4 autophosphorylation and trans-phosphorylation of IRAK1 and IRAK2 promote several transcription factors including NF-κB, interferon regulatory factor 5 (IRF5), activation protein 1 (AP-1), and cAMP response element binding protein (CREB) to induce expression of the key mediators of inflammation such as IL-6, IL-8, monocyte chemoattractant protein 1 (MCP-1), and cyclooxygenase 2 (COX-2) (24). Unlike IL-1R1, IL-1R2 lacks the TIR domain and signal-transducing properties despite binding of IL-1α/β and IL-3. Thus, IL-1R2 functions as a decoy factor limiting the pro-inflammatory effects of IL-1β or IL-1α in a competitive manner. Also in contrast to the broad IL-1R1 distribution in a wide variety of cell types, constitutive expression of IL-1R2 is rather restricted to monocytes, neutrophils, and B cells preserving these immune cells from exaggerated pro-inflammatory responses (25). Inducible IL-1R2 expression has been further reported in keratinocytes, endothelial cells, and T cells. In fact, several lines of evidence suggest that IL-1R1 signaling is crucial for priming of the pro-inflammatory T helper 17 cells (Th17), whereas induction of IL-1R2 in Th17 and regulatory T cells (Tregs) dampens their responses to IL-1 (26–29). Therefore, modulation of IL-1R2 vs. IL-1R1 surface abundance contributes to the fine tuning of immune responses. Moreover, IL-1R2 exists in a soluble form (sIL-1R2) generated either by alternative splicing or proteolytic cleavage (30, 31). Notably, the binding affinity of IL-1R2 to IL-1β is substantially higher than to IL-1α or IL-1Ra, whereas the dissociation rate for IL-1β is slower compared to IL-1α or IL-1Ra (32, 33). Relative high normal plasma levels of sIL-1R2 ranging from 5 to 10 ng/ml imply a sufficient buffering capacity for IL-1β in health (24). The ability of sIL-1R2 to trap secreted IL-1β or IL-1α may be further enhanced by the soluble IL-1R3 variant (sIL-1R3) derived from alternative splicing. Binding assays suggest that the interaction between sIL-1R2 and sIL-1R3 markedly increases the receptor affinity to IL-1α and IL-1β but not to IL-1Ra (34).

### Renal expression of IL-1 signaling networks in health and disease

The significance of IL-1 signaling in kidney disease has been increasingly recognized but the data on renal expression and distribution of its components remain scarce and partially controversial. IL-1α is typically enriched in epithelia constituting the barrier function. Consistent with this, relative high IL-1α levels were detected in the lower urinary tract and their alterations have been discussed in the context of urinary bladder cancer and urinary tract infections (35, 36). Although constitutive IL-1α expression has been postulated for a broad spectrum of epithelial cells including the renal tubular cells, no convincing localization studies are available for the kidney to our knowledge (15). However, the IL-1α expression may be low in healthy kidney tissue but induced in disease as has been shown in kidney biopsies from patients with diabetic kidney disease (DKD) and cultured renal proximal tubule cells exposed to high glucose stress (37). Likewise, induction of IL-1β in response to uropathogenic Escherichia coli (UPEC) was documented in renal fibroblasts (38). To our knowledge, no IL-1β expression was documented in kidney epithelial cells at steady state or upon challenge except of glomerular IL-1β mRNA induction in a rat model of glomerulonephritis (39). In line with this, formation of NLRP3 inflammasomes required for proteolytic processing of the IL-1β precursor has been convincingly documented in renal macrophages and dendritic cells, whereas their presence in non-immune kidney cells remains debatable (40, 41). Similar to IL-1β, evidence for renal expression of IL-1Ra in non-immune cells is restricted to glomerular detection in a rat glomerulonephritis model (39). Finally, IL-1R1 expression was recorded in glomeruli and shown to be essential for podocyte survival upon pathophysiological challenge (42). Notably, a systematic mapping of IL-1R1 distribution in mouse tissues using IL-1R1 reporter mice revealed no detectable IL-1R1 mRNA or protein in the kidney despite amplification techniques applied (43). With respect to IL-1R2, available data suggest that expression of this receptor type is induced in clear cell renal cell carcinoma (RCC) cells with implications in disease progression (44). Moreover, alterations in renal IL-1R2 expression were reported in association with multiple kidney pathology including chronic kidney disease (CKD), acute kidney injury (AKI), lupus nephritis, IgA nephropathy, RCC, and rhabdoid kidney tumor (45). Despite the apparent major role of IL-1R2 in kidney pathology, no systematic localization studies on IL-1R2 expression and distribution in mammalian kidney have been performed to our knowledge. The available data on expression and distribution of IL-1 signaling components in mammalian kidney are summarized the **Table 1**.

**Table 1:** Expression of IL-1 signaling components in the kidney and urinary bladder.

Expression	IL-1a	IL-1b	IL-Ra	IL-1R1	IL-1R2	Ref.
Kidney						
Glomerulus	+ (h)	+ (h, r)	+ (r)	+ (h, m)		(39, 42, 46)
Tubules	+ (h)			+ (h)	+ (h)	(37, 46)
Vasculature					+ (h)	(46)
Interstitium		+ (h)			+ (h)	(38, 46)
Urinary tract						
Urinary bladder	+ (h)	+ (h)	+ (h)			(35, 47)

Reported expression (+), human (h), mouse (m), rat (r), reference (Ref.)

### Effects of IL-1 in the kidney

IL-1 has been shown to induce natriuresis and diuresis in rodents via stimulation of renal cyclooxygenase 2 (COX-2) activity leading to increased bioavailability of COX-2-derived prostanoids such as prostaglandin E2 (PGE2). In the most conducted studies, natriuretic and diuretic effects of IL-1β could be blunted or abolished by unselective or selective COX-2 inhibitors suggesting a causal relationship between IL-1β and renal COX activity (48–53). The natriuresis was attributed to inhibition of tubular sodium reabsorption rather than alterations in the glomerular filtration rate (GFR) (48, 49, 54). In general, stimulatory effects of IL-1α and IL-1β on COX-2 were well documented in different cell types and tissues suggesting a similar action in the kidney (55–58). IL-1-dependent induction of COX-2 in cultured renal mesangial cells could be prevented by a calcineurin inhibitor (CNI) cyclosporin A implicating calcineurin in the IL-1 signal transduction (59). Notably, CNI are widely used for immunosuppression in organ transplantation but their therapeutic action is limited by nephrotoxicity in part due to COX-2 suppression (60).

While the aforementioned work reports diuretic and natriuretic effects of IL-1, contrasting results suggesting renal salt retention in response to IL-1R1 stimulation were provided by a study in rats with angiotensin II (AngII)-induced hypertension (61). This study identified renal macrophages as mediators of the cytokine effects on tubular salt reabsorption in the thick ascending limb thus stressing complex interactions between immune and non-immune cells in the renal physiological performance.

IL-1 has further been shown to increase permeability of the glomerular filtration barrier to large molecules, an effect largely mediated by generation of reactive oxygen species (ROS) in response to the cytokine (62). Thus, exaggerated IL-1 signaling may cause or aggravate proteinuria in pathophysiological settings. The glomerular filtration rate (GFR) seems to be unaffected by acute or subchronic IL-1β administration to normal rats (48, 53), while impact of the cytokine on GFR in kidney disease may be multifactorial and requires further investigations.

**Figure 2:**
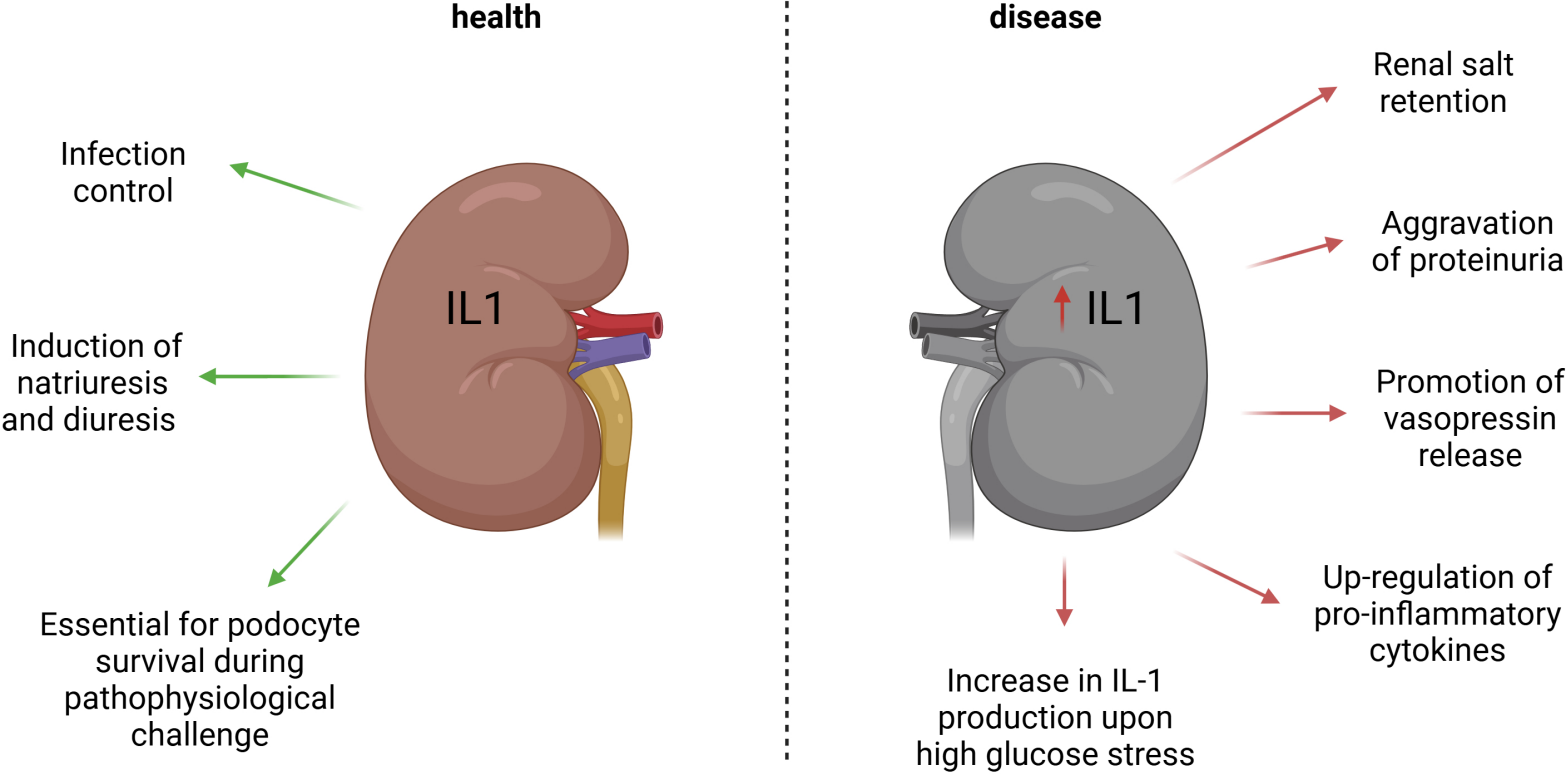
Physiologic and pathophysiologic effects of IL-1 signaling in the kidney. The left panel summarizes available data on physiological effects of the IL-1 signaling in the kidney including the coordinated antimicrobial immune response, natriuretic and diuretic actions, as well as potential contribution to the podocyte survival upon stress. The right panel illustrates pathophysiological implications of the IL-1 in kidney disease such as renal salt retention, aggravated proteinuria, amplification of vasopressin signaling, as well as local and systemic pro-inflammatory effects.

Apart from local effects in the kidney tissue, IL-1 may affect kidney performance via modulation of the endocrine hypothalamic-pituitary-adrenal (HPA) axis (63–68). IL-1 has been shown to promote vasopressin release most likely via direct effects on vasopressin-producing hypothalamic neurons (69–72). Vasopressin, in turn, may support the natriuretic while limiting the antidiuretic effects of the cytokine via distinct vasopressin receptor types (73). In addition, the vasoconstrictive action of vasopressin may blunt the vasodilating effect of IL-1β and their net effect on the renal vasculature depends on the local availability of prostaglandins (74–76). Finally, chronically elevated circulating vasopressin levels exert unfavorable effects on the kidney (77). The spectrum of potential physiological and pathophysiological IL-1 effects in the kidney is presented in **Figure 2**.

### Pharmacological modulation of IL-1 signaling

**Figure 3:**
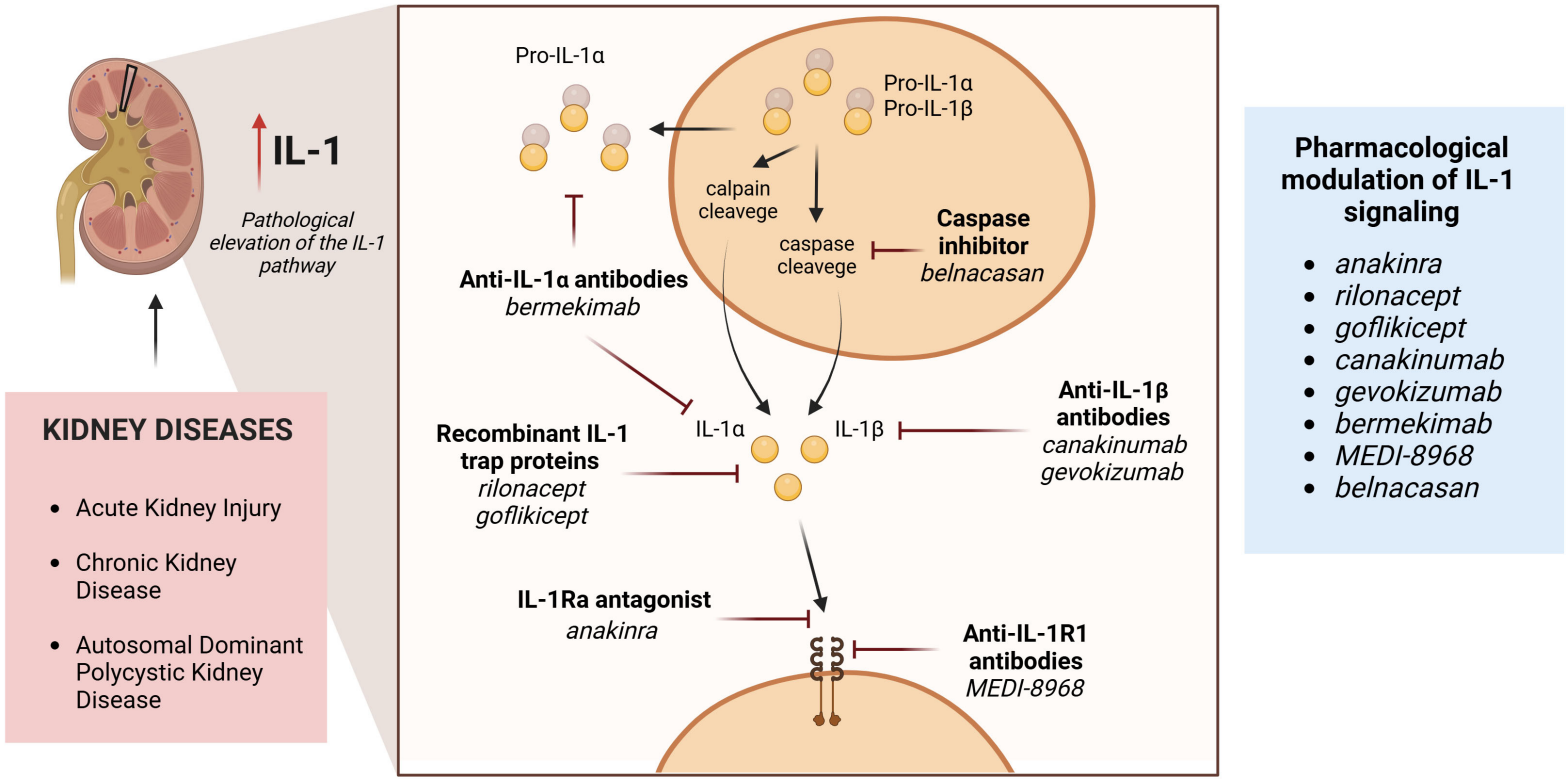
Potential nephrological fields for therapeutic application of distinct IL-1 signaling inhibitors and mechanisms of actions. The left panel describes the large renal pathologies which may be responsive to IL-1 signaling inhibitors. The middle panel illustrates mechanisms of IL-1 signaling suppression utilized by distinct approved inhibitor drugs. The right panel provides the list of currently available and clinically approved IL-1 inhibiting drugs.

Several IL-1 signaling inhibitors with distinct mechanisms of action have been clinically approved (78). The modified recombinant IL-1Ra antagonist *anakinra* competes with both IL-1α and IL-1β for the binding to IL-1R thus suppressing their signal transduction. The recombinant IL-1 trap proteins *rilonacept* and *goflikicept* contain the extracellular IL-1R1 portion enabling them to compete with IL-1R1 for the binding of IL-1β, IL-1α, or IL-1Ra. The neutralizing antibodies to IL-1β (*canakinumab*, *gevokizumab*), IL-1α (*bermekimab*), or IL-1R1 (*MEDI-8968*) have been developed as well. Finally, small molecule inhibitors of caspase 1 (*belnacasan*) prevent IL-1β maturation (**Figure 3**). The therapeutic application field for IL-1 inhibiting drugs encompasses a broad spectrum of local and systemic (auto)inflammatory disorders primarily affecting joints and bones with reported complications in other organs including kidneys. A wide range of cardiovascular and metabolic disorders including gout, post-myocardial infarction remodeling, cerebrovascular accident, diabetes mellitus, and metabolic syndrome may profit from IL-1 inhibiting therapies as well (78). With respect to the nephrological implementation, different settings of the acute kidney injury and chronic kidney disease have been suggested by experimental studies, whereas the clinical data is still limited (**Figure 3**).

### IL-1 inhibition in kidney diseases

Sterile inflammation with ensuing immune-mediated kidney damage is a hallmark of many acute and chronic kidney diseases. Disinhibition of IL-1 signaling triggers local inflammation of renal tissue and may initiate or aggravate systemic inflammatory reactions as well. The endogenous IL-1 suppressors, IL-1Ra, IL-1R2, buffer the excessive activity of IL-1α and IL-1β thus restricting and modulating the inflammatory responses. Progression of kidney diseases is typically associated with a disbalance between the pro-inflammatory and anti-inflammatory IL-1 signaling components. In this context, pharmacological inhibitors of IL-1β receive increasing attention as tools bearing potential to alleviate immune-mediated kidney damage and preserve the functional renal architecture.

## Acute Kidney Injury

Acute Kidney Injury (AKI) is a clinical syndrome characterized by an abrupt significant decline of renal function manifesting within several hours up to several days (79). AKI is prevalent in patients receiving intensive care and associated with high risk of mortality and substantial financial burden (80). Hospital-based epidemiological studies report the AKI incidence in the range of 1 in 5 adults and 1 in 3 children during a hospital episode of care worldwide (81). AKI frequently develops sudden, which is a great challenge for rapid implementation of targeted etiologic therapy. Hypovolemia, nephrotoxic medications, ischemia, acute urinary tract obstruction, and acute glomerulonephritis belong to the most common AKI causes (79). Independently on the primary cause, AKI is associated with the onset of sterile inflammation in the kidney tissue due to damage and death of renal cells through apoptosis or necrosis (82). Experimental studies on the role of inflammation during AKI and ensuing kidney damage suggest that dampening maladaptive inflammatory response may support the intrinsic regeneration of kidney epithelia thus reducing the nephron loss (83).

IL-1β has been increasingly recognized as an emerging target in AKI due to its major role in triggering local and systemic inflammatory reactions. Consequently, therapeutic potential of IL-1β inhibition has been investigated in rodents and cultured cells. Modeling of cisplatin-induced AKI in normal vs. global IL-1R1 knockout mice (IL-1R1-/-) revealed a milder kidney injury in the IL-1R1-/- strain suggesting that blockade of IL-1 signaling may exert renoprotective effects in AKI induced by nephrotoxic agents (84). In contrast, selective deletion of IL-1R1 in myeloid cells exacerbated kidney damage in kidney ischemia/reperfusion (I/R) injury model of AKI suggesting that IL-1 signaling in myeloid cells may act as a negative feedback loop to dampen excessive inflammation (85). Indeed, activation of IL-R1 in myeloid cells has been shown to promote IL-Ra expression and release that may reduce the pro-inflammatory IL-1β signal in renal tubular cells, as well as in IL-1β producing immune cells. Thus, exaggerated IL-1β production and release by IL-1R1-deficient myeloid cells in response to multiple pro-inflammatory stimuli other than IL-1β aggravated the kidney damage in mouse AKI model (85). Despite apparently discrepant AKI outcomes in global vs. myeloid cell-specific IL-1R1 knockout mouse strains, both models provide conceptual support for pharmacological suppression of IL-1 signaling in AKI. Clinical application of the IL-1R1 antagonist anakinra in combination with zinc to treat severe alcoholic hepatitis was associated with higher AKI incidence, more severe AKI course, and lower overall survival compared to prednisolone (86). However, this fact does not directly point to nephrotoxicity of anakinra but rather reflects lower anti-inflammatory efficiency of anakinra compared to prednisolone in the setting of strong systemic inflammation and multiorgan toxicity due to severe liver failure. The coronavirus 19 (COVID-19) infection is another condition frequently complicated by AKI. Several lines of evidence suggest that the severe acute respiratory syndrome coronavirus 2 (SARS-CoV-2) may directly damage the kidney epithelia in addition to the hypoxia and systemic inflammation (87). Experiments in an *ex vivo* human cellular model identified IL-1 signaling as a mediator of SARS-Cov2 renal injury and suggested that IL-1 inhibitors may ameliorate the kidney damage (87). Although clinical experience with IL-1 inhibitors for managing the SARS-Cov2-induced hyperinflammatory response is limited, benefits for survival of critically ill COVID-19 patients with hyperinflammation have been reported (88). Moreover, two studies claimed a superior effect of the IL-1R1 inhibitor anakinra compared to an IL-6 receptor inhibitor tocilizumab in managing the SARS-Cov2 hyperinflammation (89, 90). Interestingly, higher circulating levels of IL-1Ra in COVID-19 patients experiencing AKI have been associated with a better prognosis thus indirectly suggesting benefits of IL-1 inhibition (91). In contrast, a Cochrane-based meta-analysis of six randomized controlled studies of IL-1 inhibitors (anakinra or canakinumab) revealed no evidence for relevant benefits of the IL-1 inhibiting strategy in COVID-19 patients although AKI incidence and outcomes were not specifically assessed by the meta-analysis (92). Taken together, the available clinical research provides no convincing evidence in support of anti-IL-1 therapy in patients with AKI, at least in comparison to the standard care.

## Chronic Kidney Disease

Chronic Kidney Disease (CKD) is a syndrome of gradual kidney function loss defined as a chronic GFR decline (<60 ml/min*1,73m^2^) or albuminuria (>30 mg/g creatinine) persisting for three months or longer thus reflecting an irreversible deterioration of kidney function (93). CKD progression is associated with accumulation of toxic metabolites in the body triggering secondary multiorgan damage and systemic inflammation (94). Because of the major impact of the kidney on the fluid homeostasis and blood pressure control, limitations of renal functions principally affect cardiovascular performance. Suppression of IL-1β using canakinumab has been shown to decrease the incidence of major adverse cardiovascular effects in CKD patients with advanced atherosclerosis (95). Similarly, administration of the IL-1α/β inhibitor rilonacept reduced systemic inflammation and improved vasodilatory arterial function in CKD patients (96). The individual roles of IL-1α vs. IL-1β in the setting of acute myocardial infarction (AMI) and CKD were investigated in patients and knockout mice deficient for IL-1α vs. IL-1β (97). Interestingly, IL-1α but not IL-1β appears to play a major role in leukocyte-endothelial adhesion and vascular inflammatory injury driving the cardiovascular complications, as well as CKD progression. These pathophysiological effects may be mediated via juxtacrine IL-1α signal transduction from monocytes to endothelial cells (97). Importantly, IL-1α knockout mice were largely protected against oxalate- or adenine-induced kidney damage, which stresses the pathophysiological role of IL-1α in CKD (97). Another experimental study in IL-1Ra-deficient mice implicated exaggerated IL-1β signaling in progression of CKD and anemia by showing that an antibody targeting IL-1β was able to ameliorate the kidney damage and improve the response to hypoxia (98).

In addition to the experimental and clinical data, genetic studies in human revealed strong links between certain polymorphisms within the IL-1 gene cluster and risk of the End Stage Renal Disease (ESRD) (99, 100). Blunted negative feedback response to inflammation via IL-1Ra production has been associated with renal involvement in autoimmune diseases (101). A pilot clinical trial of anakinra in ESRD patients receiving hemodialysis suggested that IL-1R1 antagonism may help to reduce systemic inflammation and is safe in this clinical setting (102). Further studies in patients on hemodialysis maintenance corroborated the anti-inflammatory effect of anakinra accompanied by enhanced levels of adiponectin, although expected metabolic adiponectin effects such as normalization of insulin sensitivity or protein metabolism were not observed (103–105). Anakinra has been further tested in porcine kidneys subjected to renal normothermic machine perfusion. The resulting downregulation of IL-6 and other pro-inflammatory genes points to a potential of anakinra to ameliorate the immune-mediated transplant kidney injury (106). Mendelian randomization revealed a significant positive correlation between serum IL-1Ra levels and GFR values in CKD cohorts suggesting that dampening the IL-1 signaling by IL-1Ra is renoprotective (107). Suppression of IL-1β has been further shown to attenuate CKD progression in obese and diabetic db/db mice as reflected by milder GFR decline and reduced renal expression of kidney damage biomarkers including the neutrophil gelatinase-associated lipocalin (NGAL) (108, 109). Notably, experiments in mice have shown that IL-1β is a strong inducer of NGAL expression (110). Elevated circulating and renal NGAL levels in response to subcutaneous IL-1β infusion in mice were not accompanied by an overt kidney damage suggesting that enhanced renal and urinary NGAL levels observed in patients may be partially related to IL-1β induction rather than to the degree of renal tissue damage (110). Indirect suppression of IL-1β signaling by a caspase 1 inhibitor belnacasan (also known as VX-765) has been shown to ameliorate kidney damage and fibrosis in experimental AKI and CKD models but these beneficial effects may be also related to inhibition of pyroptosis (111–113).

Finally, the IL-1β inhibitors anakinra and canakinumab have proven marked efficiency in treatment of renal amyloidosis in patients with Familial Mediterranean Fever (FMF) (114). FMF is the most common inherited autoinflammatory disease distinguished by recurrent attacks of fever and serositis frequently complicated by secondary renal amyloidosis (~8-9% of patients) with rapid progression to ESRD (115). In the most patients, FMF is caused by hypermorphic bi-allelic mutations in the Mediterranean Fever gene (*MEFV*) encoding Pyrin, which decrease the threshold for formation of Pyrin inflammasomes with ensuing hyperactivation of IL-1β processing and secretion (116, 117). FMF therapy is based on colchicine, but 5-10% of patients show weak or no response to this medication and are at particularly high risk of developing renal complications. Importantly, IL-1 inhibiting strategy demonstrated high efficiency in achieving complete disease remission, as well as improvement or stabilization of kidney function in colchicine-resistant FMF patients with renal amyloidosis (114, 115, 118). However, a case series study in pediatric patients with secondary amyloidosis due to autoinflammatory diseases treated with IL-1 inhibitors anakinra or canakinumab reported no decrease or even enlargement of amyloid deposits in kidney tissue despite reduction of proteinuria (119). In contrast, another small cohort analysis of hemodialysis patients empirically treated with IL-1 inhibitors reported stabilization or reduction of renal amyloid load as detected by serum amyloid-P scintigraphy (120). Finally, isolated cases of anakinra-associated renal amyloidosis were reported recently (121, 122). Thus, anakinra may cause subcutaneous amyloidosis at the site of injection, as well as renal or systemic amyloidosis (123). This fact has been recognized in a recent update of the amyloid nomenclature (124). IL-1β neutralizing antibodies may represent a safer alternative in this respect. Moreover, early initiation of canakinumab before decline of GFR below 60 ml/min*1,73m^2^ appears mandatory for efficient reduction of proteinuria in FMF patients with renal amyloidosis (125).

## Autosomal dominant polycystic kidney disease

IL-1 signaling may contribute to pathophysiology of the autosomal dominant polycystic kidney disease (ADPKD) as suggested by enhanced IL-1α and IL-1β expression in kidneys specimens from ADPKD patients, presence of IL-1β in the cystic fluid, decreased urinary IL-1Ra excretion in ADPKD patients, kidney disease-related polymorphisms within the IL-1 gene cluster, as well as protective effects of genetic IL-1R1 deletion in an ADPKD mouse model (99, 100, 126–128). Being the most prevalent inherited kidney disease affecting up to 1 of 500 people, ADPKD represents a significant burden due to challenging conservative management and frequent progress to ESRD requiring kidney replacement therapy (129). In most affected individuals, ADPKD is caused by inactivating mutations in the PKD1 or PKD2 genes encoding for polycystin 1 (PC1) and polycystin 2 (PC2), respectively (130). PC1 and PC2 build a molecular complex with transient receptor potential (TRP) channel activity which plays a critical role in the functionality of primary cilium (131). The disease pathophysiology is attributable to reduced polycystin signaling in the primary cilium due to mutations in *PKD1*, *PKD2*, or some other genes involved in maturation and trafficking of the PC1/PC2 complex (130). Molecular details of cysts induction are still poorly understood. Elevation of the intracellular cAMP has been meanwhile established as a factor promoting proliferation of cyst epithelium and cyst growth (132). Disrupted intracellular calcium signaling caused by PKD gene mutations enables disproportionally strong cAMP-induced activation of the Mitogen-Activated Protein Kinase/Extracellular Signal-Regulated Kinase (MEK or MAPKK) and the downstream Extracellular Signal-Regulated Kinase (ERK), promoting epithelial proliferation and cyst growth in ADPKD (133). Additionally, elevated cAMP levels stimulate activity of the cystic fibrosis transmembrane conductance regulator (CFTR) chloride channel and ensuing transepithelial chloride secretion driving accumulation of water in cysts (133). For these reasons, the cAMP-generating hormone vasopressin acting in the renal collecting duct via its V2 receptor aggravates the cyst growth, whereas selective V2 receptor antagonists (vaptans) retard ADPKD progression (134). IL-1R1 activation recruits the cAMP signaling pathway either directly or via PGE2 thus enhancing intracellular cAMP levels as well (135–138). The Mammalian Target of Rapamycin (mTOR) signaling is another potential pathway linking IL-1 to ADPKD progression. The mTOR signaling pathway is essential for protein translation, cell growth and proliferation in the physiological setting, whereas excessive mTOR activity has been implicated in pathophysiology of renal cyst formation in ADPKD and tuberous sclerosis (139–142). Several physiological and pathophysiological effects of IL-1β in the kidney are mediated by mTOR activation. While the activation of the IL-1R1 and downstream stimulation of the mTOR signaling in podocytes is cytoprotective, renal tubular cells exhibit pro-inflammatory and pro-fibrotic responses instead (42, 143, 144). Although transgenic or pharmacologic mTOR inhibition alleviated ADPKD in experimental animal studies, the clinical success of mTOR inhibitors (sirolimus and everolimus) was rather limited in ADPKD patients (145–148). The inefficiency of mTOR inhibitors may be related to lower dosing in the clinical ADPKD setting as compared to experimental conditions, since these drugs may cause serious systemic adverse effects (149). Likewise, long-term therapy with V2 receptor antagonists such as tolvaptan to lower intracellular cAMP levels and retard proliferation rate in cyst epithelium is limited by polyuria and hepatic adverse effects in the real clinical situation (150). The scarcity of therapeutic interventions in ADPKD produces a strong demand for new options. Inhibitors of the IL-1 signaling may help to retard the ADPKD progression by combined suppression of cAMP and mTOR signaling pathways. In addition to direct effects in the cyst epithelium, inhibition of IL-1 cytokines may reduce inflammation-driven ADPKD complications such as hypertension or insulin resistance (151, 152). Further mechanistic elucidation of the IL-1 signaling in the ADPKD environment is mandatory to assess the therapeutic potential of available IL-1 inhibitors.

### Limitations of IL-1 inhibitors in renal patients

#### Safety

Accumulating clinical data suggests that IL-1 inhibiting agents are generally safe and well tolerable in patients with mild, moderate, or even advanced CKD. Moreover, suppression of IL-1 signaling provides cardiovascular benefits in CKD patients who are otherwise at increased risk of cardiovascular events (CVE) due to systemic inflammation and vascular problems associated with CKD progression (94, 95). The CANTOS trial (Canakinumab Anti-Inflammatory Thrombosis Outcome Study) enrolled 10061 patients with a history of prior myocardial infarction and elevated high-sensitivity C-reactive protein (hsCRP) including 1875 patients with CKD stages 1–3 thus providing a solid evaluation platform for renal safety of canakinumab in the setting of mild to moderate CKD (95). Retrospective analysis of CKD patients enrolled in the CANTOS study revealed no relevant safety problems with canakinumab (95). With respect to the advanced CKD (stages 4-5), no major safety concerns have been identified by pilot and retrospective clinical studies with anakinra or canakinumab. A retrospective analysis of 31 patients with advanced CKD or kidney transplantation receiving anakinra for management of gout revealed only one serious infection which was unrelated to the anakinra therapy (153). Similarly, a pilot study of anakinra in two hemodialysis patients with pseudo-arthritis caused calcium pyrophosphate deposition demonstrated high efficacy and very good safety at long term (154). In addition, a recent retrospective analysis of anakinra and canakinumab in kidney transplant recipients suffering from FMF demonstrated prolonged graft survival and lower rejection supporting renal benefits of IL-1 inhibition (155). However, the anti-IL-1 therapy was associated with higher mortality rate among kidney transplant recipients with FMF due to infections or unknown reasons (155). Since organ transplantation is accompanied by a strong immunosuppressive therapy, additional immunosuppression provided by IL-1 inhibiting agents is a critical point of concern in kidney transplant recipients independently on renal benefits. Therefore, guidelines for adjustment of standard immunosuppressive protocols to supplementary anti-IL-1 therapy need to be established to prevent serious infections in organ transplant recipients. In view of recent information on iatrogenic renal or systemic amyloidosis rarely associated with anakinra, an initial choice of or switch to canakinumab might be considered in kidney transplant recipients to ensure maximal renal safety of IL-1 inhibition (118, 121–124).

#### Cost effectiveness

Broad adoption of IL-1 inhibiting strategies in non-orphan kidney diseases such as CKD may face significant cost-efficiency challenges due to the high patient numbers and chronic disease course (156). The CANTOS study rendered canakinumab not cost-effective for prevention of recurrent CVE and the situation with CKD may be largely the same (157). Similarly, long-term anakinra therapy is associated with extremely high costs (158). Genetic biomarkers predicting high response to IL-1 inhibition such as polymorphisms within the IL-1 gene cluster may provide reasonable therapeutic niches for IL-1 inhibiting agents with improved cost-efficiency in the context of ESRD and kidney transplantation (99, 100). ADPKD may represent another cost-effective model if clinical evidence corroborates the putative therapeutic potential of IL-1 suppression suggested by experimental and genetic studies (99, 100, 126–128).

### Current state and future perspectives

Based on the available research and clinical information, IL-1 inhibitors may be of advantage in colchicine-resistant FMF patients with secondary renal amyloidosis. IL-1β neutralizing antibodies such as canakinumab may be safer compared to IL-1Ra analogues such as anakinra, since the latter may induce or aggravate amyloid deposits in rare cases (114, 115, 118, 122, 123). Management of FMF in kidney transplant recipients using anti-IL-1 agents has proven efficiency but revealed safety problems potentially related to immunosuppressive drug effects requiring adjustment of standard immunosuppressive regimen in such patients (155). IL-1α or IL-1β suppression reduces cardiovascular risks in patients with late CKD stages, including ESRD and/or prolonged hemodialysis, but cost-effectiveness limits broad implementation of these strategies (95, 96, 157). IL-1 inhibitors may further contribute to improved management of hyperinflammation and kidney damage associated with SARS-Cov2 but supporting clinical evidence remains limited and partly controversial. ADPKD may emerge as a therapeutic niche for IL-1 inhibiting agents especially in the light of scarcity of currently available strategies to delay cyst growth. Clinical trials of IL-1 inhibiting agents in ADPKD patients are pending to validate the experimental data (126–128). In general, selective pharmacological suppressors of pro-inflammatory pathways, such as IL-1 signaling inhibitors, hold great promise for improved management of local and systemic inflammation in diverse nephrological conditions (**Table 2**). However, an improved mechanistic understanding of their effects in the kidney is mandatory to identify reasonable therapeutic niches balancing benefits with risks and cost effectiveness. Recent progress in omics-based analytics led to development of the high-plex protein and whole transcriptomics co-mapping technology integrating proteomic and transcriptomic data at spatial resolution (159). Search for optimal nephrological indications for IL-1 inhibiting agents would strongly profit from implementation of such multi-modal, omics-based, spatial-resolving methods permitting detailed molecular characterization of cellular and tissue drug effects in kidney samples from animal models or patient biopsies.

**Table 2:** Summary of potential renal indications for and clinical experience with interleukin-1 inhibiting agents.

Disease	IL-1 signaling	Tested IL-1 inhibitors (effects)	References
AKI	↑IL-1β	anakinra (~)	(86)
COVID-19/AKI	↑IL-1β	anakinra (+, ~)	(88–90, 92)
CKD (1–3)	↑IL-1α/IL-1β	canakinumab (+), rilonacept (+)	(95, 96)
ESRD/HD	↑IL-1α/IL-1β	anakinra (+)	(153, 154)
KT	↑IL-1α/IL-1β	anakinra (+, !), canakinumab (+, !)	(155)
FMF/renal AA	↑IL-1β	anakinra (+, !), canakinumab (+)	(114, 115, 118, 121)
ADPKD	↑IL-1α/IL-1β	no clinical information	(126)

Acute kidney injury (AKI), chronic kidney disease (CKD), end stage renal disease (ESRD), hemodialysis (HD), kidney transplantation (KT), Familial Mediterranean Fever (FMF), serum amyloid A protein amyloidosis (AA), autosomal dominant polycystic kidney disease (ADPKD), increased levels of IL-1 isoforms in plasma or cystic fluid (↑), proven efficacy (+), lack of significant efficacy (~), safety concerns (!).
